# Anesthetic Management of a Patient With Myasthenia Gravis Undergoing Thoracoscopic Thymectomy: A Case Report and Literature Review

**DOI:** 10.7759/cureus.116162

**Published:** 2026-09-13

**Authors:** Rufaidah Alchuban, Abdulrahman AlSulaiti, Khadija Abuhassan, Vishal Shah

**Affiliations:** 1 Anaesthesia and Pain Management Department, Royal Medical Services, Muharraq, BHR

**Keywords:** autoimmune disease, general anesthesia, myasthenia gravis (mg), respiratory muscle weakness, thoracoscopic thymectomy

## Abstract

Myasthenia gravis is an autoimmune neuromuscular disorder characterized by fluctuating skeletal muscle weakness and fatigability. Perioperative management is challenging because patients may have bulbar or respiratory muscle involvement and altered sensitivity to neuromuscular blocking agents, increasing the risk of postoperative respiratory failure and myasthenic crisis. Thymectomy is an established therapeutic option in selected patients with myasthenia gravis and may be performed using minimally invasive thoracoscopic techniques. We report the anesthetic management of a 48-year-old man with generalized acetylcholine receptor (AChR) antibody-positive myasthenia gravis (MGFA class IIb) scheduled for thoracoscopic thymectomy. The patient had a recent relapse with diplopia, dysphagia, and easy fatigability, which improved after medical therapy. Preoperative optimization included intravenous immunoglobulin therapy for four days. General anesthesia was induced with propofol and remifentanil without initial neuromuscular blockade. Tracheal intubation was facilitated using video laryngoscopy after topical lidocaine. Anesthesia was maintained with sevoflurane, and invasive monitoring was established. Because of the surgical requirement for improved thoracoscopic visualization, a reduced dose of rocuronium was administered intraoperatively under train-of-four monitoring. At the end of surgery, neuromuscular blockade was reversed with sugammadex, and the patient was successfully extubated and transferred to the intensive care unit in stable condition. This case highlights the importance of careful preoperative optimization, avoidance of neuromuscular blocking agents when possible, quantitative neuromuscular monitoring, and individualized use of low-dose rocuronium with sugammadex reversal when muscle relaxation is required during thoracoscopic thymectomy in patients with myasthenia gravis.

## Introduction

Myasthenia gravis (MG) is an autoimmune disorder of neuromuscular transmission characterized by fluctuating skeletal muscle weakness and fatigability. The disease is most commonly mediated by autoantibodies directed against postsynaptic acetylcholine receptors at the neuromuscular junction, although other antibody subtypes may also occur. Clinically, MG may involve ocular, bulbar, limb, axial, and respiratory muscles, with symptoms that typically worsen with exertion and improve with rest. Bulbar and respiratory muscle involvement are particularly important in the perioperative setting because they increase the risk of aspiration, ineffective cough, postoperative respiratory failure, and myasthenic crisis [1,2].

The thymus plays an important role in the pathophysiology of MG, and thymic abnormalities, including thymic hyperplasia and thymoma, are frequently associated with the disease. Thymectomy is an established therapeutic option in selected patients with MG, particularly in patients with thymoma and in appropriately selected patients with generalized acetylcholine receptor antibody-positive disease. Current international consensus guidance supports thymectomy as part of individualized MG management, taking into consideration disease severity, antibody status, thymic pathology, age, comorbidities, and response to medical therapy [2].

Thoracoscopic thymectomy has increasingly been used as a minimally invasive alternative to open surgical approaches. Compared with sternotomy, video-assisted thoracoscopic surgery may reduce surgical trauma and facilitate postoperative recovery, but it still presents important anesthetic challenges. These include the need to maintain adequate ventilation, optimize surgical exposure, manage the physiological effects of carbon dioxide insufflation when used, and avoid perioperative factors that may worsen neuromuscular weakness [3,4].

Anesthetic management of patients with MG is challenging because of their altered response to neuromuscular blocking agents. Patients with MG are generally sensitive to non-depolarizing neuromuscular blockers and may develop prolonged weakness even at reduced doses, whereas responses to depolarizing agents can be unpredictable. Therefore, neuromuscular blocking agents should be avoided whenever possible. When muscle relaxation is necessary, reduced-dose rocuronium with quantitative neuromuscular monitoring and reversal using sugammadex has been described as an effective strategy [4].

Postoperative myasthenic crisis remains one of the most serious complications after thymectomy. It is characterized by severe bulbar or respiratory muscle weakness leading to respiratory failure and the need for ventilatory support. Systematic reviews have reported that a postoperative myasthenic crisis after thymectomy is clinically significant and associated with factors such as disease severity, respiratory or bulbar involvement, thymoma, pulmonary complications, and intraoperative blood loss [5,6]. This case report describes the perioperative anesthetic management of a patient with generalized acetylcholine receptor (AChR) antibody-positive myasthenia gravis (MGFA class IIb) undergoing thoracoscopic thymectomy. The patient had bulbar involvement manifested by dysphagia. Bulbar symptoms are well-established risk factors for postoperative myasthenic crisis. Perioperative management focused on preoperative optimization with intravenous immunoglobulin, avoidance of neuromuscular blockade during induction, cautious intraoperative use of low-dose rocuronium when required, quantitative train-of-four (TOF) monitoring, and reversal with sugammadex.

## Case presentation

A 48-year-old man with a known diagnosis of generalized AChR antibody-positive myasthenia gravis (MGFA class IIb), diagnosed 8 years earlier, was scheduled for thoracoscopic thymectomy under general anesthesia. At the pre-anesthesia clinic evaluation, he reported that his initial symptoms had been treated medically for two to three months after diagnosis, after which they resolved. More recently, he developed a relapse characterized by diplopia, dysphagia, and easy fatigability when climbing stairs. The patient was started on pyridostigmine 60 mg twice daily and prednisolone 20 mg once daily, with improvement in diplopia after one month of treatment.

On examination, the patient weighed 98 kg and was 170 cm tall. Airway assessment revealed Mallampati class II, mouth opening greater than three finger breadths, thyromental distance of three finger breadths, and adequate neck mobility. No predictors of difficult airway management were identified. Cardiovascular examination revealed normal heart sounds without murmurs, and chest auscultation demonstrated equal bilateral air entry. Neurological examination at the time of assessment showed no ptosis, diplopia, or facial muscle weakness.

Laboratory investigations, including complete blood count, serum electrolytes, and thyroid function tests, were within normal limits. Acetylcholine receptor antibody level was elevated at 0.68 nmol/L, consistent with a generalized AChR antibody-positive myasthenia gravis (MGFA class IIb) diagnosis. Chest radiography and electrocardiography were unremarkable. Chest computed tomography showed no abnormal thymic tissue or mass lesion (Figure 1). Preoperative pulmonary function tests were normal, indicating adequate respiratory reserve for surgery. However, given the patient's recent bulbar symptoms, perioperative management remained focused on minimizing the risk of postoperative respiratory complications and myasthenic crisis. For perioperative optimization, the patient was admitted and, following the recommendation of the neurology team, received intravenous immunoglobulin (IVIG) at a dose of 0.5 g/kg/day for 4 consecutive days before surgery. The patient continued his regular pyridostigmine therapy, including the morning dose on the day of surgery. Pyridostigmine was resumed postoperatively once oral intake was tolerated.

**Figure 1:**
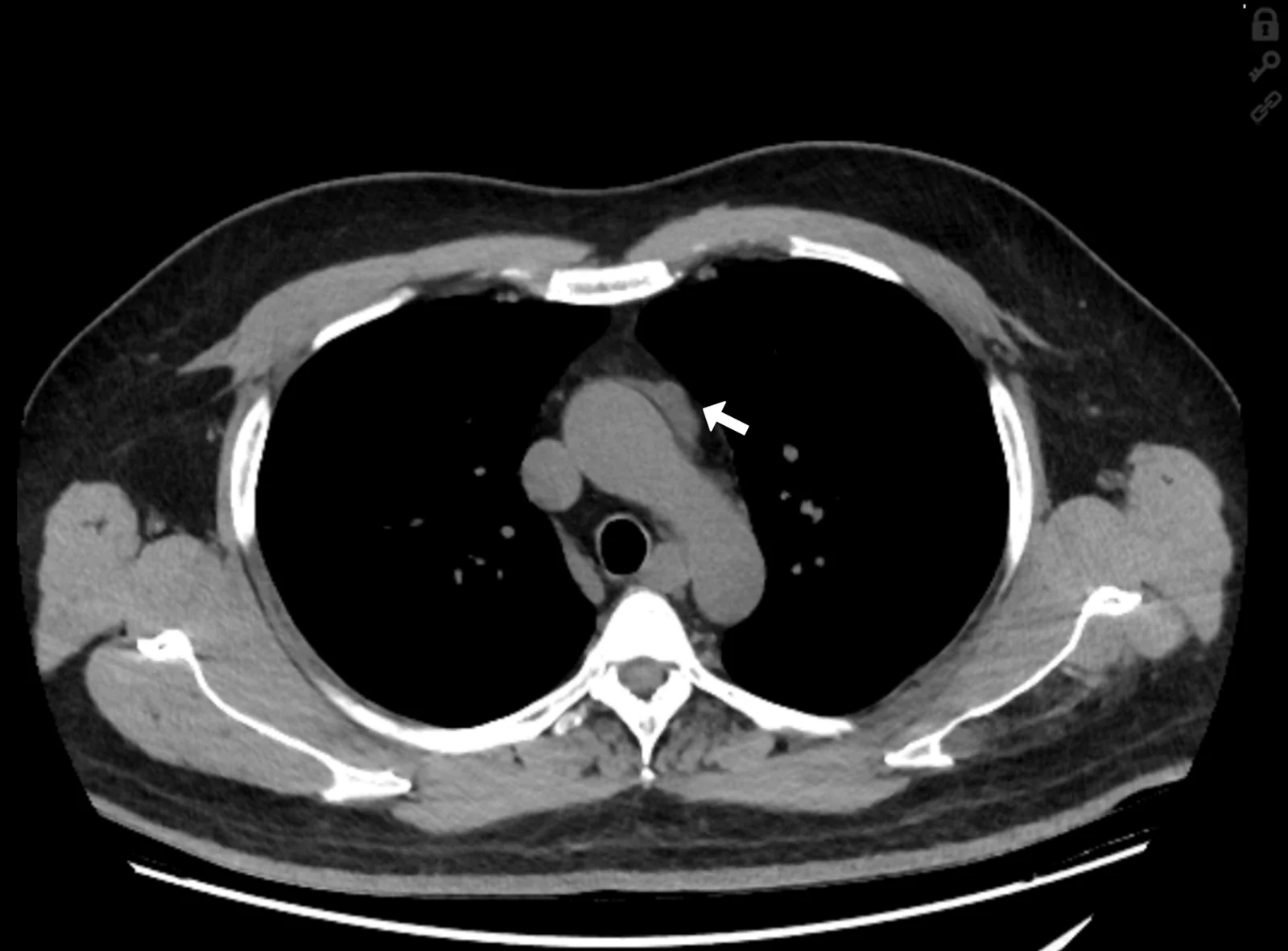
Chest computed tomography scan showing a normal thymus (arrow) and no abnormal thymic tissue or anterior mediastinal mass lesion

On the day of surgery, the patient arrived in the operating theater with a 20-gauge intravenous cannula in situ. Standard American Society of Anesthesiologists monitoring was applied, including noninvasive blood pressure, pulse oximetry, electrocardiography, and temperature monitoring. After three minutes of preoxygenation, general anesthesia was induced with remifentanil infusion at 0.05-0.1 µg/kg/min and propofol 250 mg, without initial administration of a neuromuscular blocking agent. The vocal cords were topicalized with 5 mL of 1% lidocaine to suppress airway reflexes and facilitate tracheal intubation without neuromuscular blockade. Tracheal intubation was successfully performed using a video laryngoscope, and an 8.0-mm endotracheal tube was inserted.

Intravenous hydrocortisone 100 mg was administered as perioperative stress-dose steroid coverage because the patient had been receiving prednisolone 1 month preoperatively, with concern for potential hypothalamic-pituitary-adrenal axis suppression during surgery. Intravenous paracetamol 1 g and dexamethasone 8 mg were also administered. Anesthesia was maintained with sevoflurane at a minimum alveolar concentration of 1 in 35% oxygen. A 20-gauge arterial catheter was inserted into the right radial artery, and a 7.5 Fr, 15-cm triple-lumen central venous catheter was placed in the right internal jugular vein under ultrasound guidance.

During the procedure, carbon dioxide insufflation was used to improve thoracoscopic visualization. However, because inadequate surgical exposure persisted despite insufflation, additional muscle relaxation was required. Therefore, rocuronium (0.1 mg/kg) was administered intraoperatively to optimize the surgical field. The patient remained hemodynamically stable throughout the procedure. At the end of surgery, local anesthetic infiltration was administered at the port sites. Estimated blood loss was 20 mL, and urine output was 500 mL.

Neuromuscular function was monitored using quantitative acceleromyography (TOF-Watch). Sugammadex 200 mg was administered for reversal of neuromuscular blockade. Following reversal, a TOF ratio of ≥0.9 was confirmed before tracheal extubation. Extubation was performed only after standard clinical and respiratory criteria were met. The patient was awake and able to follow commands, demonstrated adequate spontaneous ventilation, maintained satisfactory oxygenation with minimal ventilatory support, and was hemodynamically stable. He was transferred to the intensive care unit for close postoperative monitoring, particularly for signs of residual neuromuscular blockade, respiratory compromise, or myasthenic crisis. Postoperative analgesia was provided using patient-controlled analgesia with morphine. The immediate postoperative course was uneventful, with no evidence of respiratory compromise or myasthenic crisis.

## Discussion

MG presents important perioperative challenges because fluctuating skeletal muscle weakness may involve ocular, bulbar, limb, axial, and respiratory muscles. In the perioperative setting, bulbar and respiratory involvement are particularly important because they increase the risk of aspiration, ineffective cough, postoperative respiratory failure, prolonged ventilation, and myasthenic crisis. The Myasthenia Gravis Foundation of America clinical classification, originally reported by Jaretzki et al., provides a standardized framework for describing disease severity according to the distribution and severity of muscle weakness [1]. In the present case, the patient had a recent relapse characterized by diplopia, dysphagia, and easy fatigability, suggesting generalized MG with bulbar involvement before optimization. These symptoms justified careful preoperative assessment, perioperative planning, and postoperative monitoring.

Thymectomy is an established therapeutic option in selected patients with MG, particularly in patients with thymoma and in appropriately selected patients with generalized acetylcholine receptor antibody-positive disease [2]. Thoracoscopic thymectomy is increasingly used as a minimally invasive alternative to open thymectomy and may be associated with reduced surgical trauma and improved postoperative recovery [3]. However, it still creates important anesthetic challenges, including the need for optimal surgical exposure, safe airway and ventilation management, and avoidance of factors that may worsen neuromuscular weakness. In addition, rare but serious complications during video-assisted thoracoscopic thymus surgery, including major vascular injury, sudden cardiac arrest, and diaphragmatic injury, have been reported [7]. In the present case, chest computed tomography demonstrated no evidence of a thymoma or other thymic mass, and the patient was diagnosed with non-thymomatous generalized AChR antibody-positive MG. Given that thymectomy is recommended to improve long-term clinical outcomes. The patient underwent thoracoscopic thymectomy following preoperative optimization.

A key concern in MG is the altered response to neuromuscular blocking agents. Patients with MG are usually sensitive to non-depolarizing neuromuscular blockers and may develop prolonged weakness even after reduced doses, while their response to depolarizing agents may be unpredictable [4]. Therefore, neuromuscular blocking agents should be avoided whenever possible. In the present case, anesthesia was induced with propofol and remifentanil without initial neuromuscular blockade. Tracheal intubation was facilitated using video laryngoscopy after topical lidocaine, which allowed airway control while avoiding routine muscle relaxant administration during induction.

Several published reports have described successful thymectomy in patients with MG using non-muscle-relaxant techniques. Kiran et al. reported thymectomy using sevoflurane without muscle relaxants, with successful extubation and no postoperative respiratory muscle weakness [8]. El-Dawlatly described thoracoscopic thymectomy using a modified non-muscle-relaxant technique with propofol, opioid-based anesthesia, and airway topicalization, with successful postoperative extubation [9]. More recently, Hien et al. reported thoracoscopic thymectomy in MG patients using propofol target-controlled infusion or sevoflurane anesthesia without muscle relaxants [10]. These reports support the principle that thymectomy in patients with MG can often be performed without neuromuscular blockade when surgical conditions allow and when the patient has been adequately optimized.

However, complete avoidance of neuromuscular blocking agents is not always feasible, especially during minimally invasive thoracic surgery when optimal surgical exposure is required. In the present case, carbon dioxide insufflation was used to improve thoracoscopic visualization, and additional muscle relaxation became necessary intraoperatively. Therefore, a reduced dose of rocuronium was administered under quantitative acceleromyography (TOF-Watch). This reflected an individualized approach rather than routine neuromuscular blocker use. The dose was minimized, neuromuscular recovery was monitored, and sugammadex was administered before extubation.

The use of rocuronium with sugammadex has been reported in patients with MG undergoing thymectomy. Takeda et al. described successful reversal of rocuronium-induced neuromuscular blockade with sugammadex in a patient with MG undergoing thoracoscopic thymectomy [11]. Sungur Ulke et al. evaluated rocuronium and sugammadex in patients with MG undergoing thymectomy and supported the feasibility of this strategy when careful monitoring is used [12]. Uchida et al. reported that even patients with subclinical MG may show prolonged sensitivity to rocuronium, emphasizing the need for cautious dosing and quantitative neuromuscular monitoring [13]. These findings are highly relevant to the present case, in which rocuronium was avoided during induction but used later in a low dose when required, followed by sugammadex reversal.

Sugammadex is useful in this context because it reverses aminosteroid neuromuscular blockers such as rocuronium through encapsulation rather than acetylcholinesterase inhibition. This mechanism avoids the cholinergic effects and variable response associated with traditional anticholinesterase reversal. A retrospective analysis comparing sugammadex with neostigmine/glycopyrrolate after video-assisted thoracoscopic thymectomy in MG patients suggested that sugammadex may be a useful reversal strategy in this population [14]. Nevertheless, sugammadex does not eliminate the need for postoperative vigilance because respiratory failure may still occur due to underlying disease severity, bulbar weakness, pulmonary complications, or inadequate respiratory reserve.

Preoperative optimization is another important component of perioperative management. Intravenous immunoglobulin and therapeutic plasma exchange are used in selected patients with MG, particularly those with severe symptoms, bulbar involvement, respiratory weakness, or increased risk of perioperative deterioration [2,15]. In the present case, the patient received intravenous immunoglobulin at 0.5 g/kg/day for 4 days before surgery because of recent relapse symptoms, including dysphagia and easy fatigability. This strategy was used to improve perioperative stability and reduce the risk of postoperative deterioration.

Early extubation after thymectomy may reduce complications related to prolonged mechanical ventilation, including airway injury, secretion retention, atelectasis, ventilator-associated pneumonia, sedation-related complications, and prolonged intensive care stay [16]. In the present case, extubation was performed only after complete neuromuscular recovery was confirmed clinically and with quantitative TOF monitoring (TOF ratio ≥0.9), and after standard clinical and respiratory extubation criteria had been satisfied, including adequate spontaneous ventilation, satisfactory oxygenation, hemodynamic stability, and the ability to protect the airway. The patient was extubated successfully and transferred to the intensive care unit in stable condition, with no immediate evidence of respiratory compromise or myasthenic crisis.

Postoperative myasthenic crisis remains one of the most serious complications after thymectomy. It is characterized by severe respiratory or bulbar muscle weakness requiring ventilatory support. Systematic reviews have identified several risk factors, including disease severity, history of myasthenic crisis, bulbar symptoms, thymoma, pulmonary complications, and increased intraoperative blood loss [5,6]. In the present case, several protective factors were present, including preoperative optimization, minimal blood loss, cautious use of neuromuscular blockade, train-of-four monitoring, sugammadex reversal, successful extubation, and postoperative intensive care unit observation. Although sugammadex enabled effective reversal of neuromuscular blockade, it did not replace the need for vigilant postoperative monitoring, which remained essential for the early detection and management of respiratory or bulbar muscle weakness.

A summary of selected published reports on anesthetic strategies for thymectomy in patients with myasthenia gravis is provided in Table 1. The available literature shows two main anesthetic strategies: complete avoidance of neuromuscular blocking agents when surgical conditions permit, or cautious use of reduced-dose neuromuscular blockade with quantitative monitoring and appropriate reversal when muscle relaxation is required. The present case contributes to the latter approach by demonstrating that, when muscle relaxation became necessary during thoracoscopic thymectomy, low-dose rocuronium could be used safely when guided by TOF monitoring and followed by sugammadex reversal. This approach may be particularly relevant in thoracoscopic procedures requiring improved surgical exposure.

**Table 1:** Summary of selected published reports on anesthetic management of thymectomy in patients with myasthenia gravis Sources: [8-14,17,18] NMBA: neuromuscular blocking agent; MG: myasthenia gravis; NSAID: nonsteroidal anti-inflammatory drug; AChR: acetylcholine receptor; TOF: train-of-four; VATS: video-assisted thoracoscopic surgery

Study	Type	Patient/sample	Surgery	Preoperative optimization	Anesthetic strategy	NMBA/reversal	Outcome	Relevance to current case
Kiran et al. [8]	Case report	8-year-old boy with juvenile MG and thymoma	Open thymectomy via midsternotomy	Continued morning anticholinesterase dose; CT confirmed thymoma	Sevoflurane used as sole anesthetic agent; neuromuscular monitoring used; NSAIDs used to avoid the effects of narcotic analgesics	No muscle relaxant used	Rapid emergence and extubation; no postoperative respiratory muscle weakness reported	Demonstrates feasibility of avoiding muscle relaxants in MG thymectomy, although surgical approach differed from current thoracoscopic case.
El-Dawlatly [9]	Two case reports	Case 1: 23-year-old female with generalized MG, previous respiratory failure and bulbar involvement. Case 2: 21-year-old female with MG	Thoracoscopic thymectomy	Case 1 received pyridostigmine, prednisolone, and three sessions of plasmapheresis; Case 2 was on pyridostigmine/prednisolone	Modified non-muscle-relaxant technique using propofol and sufentanil; airway topicalization with lignocaine/xylocaine; DLT used	No muscle relaxant used	Both were extubated after surgery. Case 1 had postoperative right upper-lobe collapse due to secretions but recovered; Case 2 had uneventful recovery	Very similar to current case because it involved thoracoscopic thymectomy in MG and emphasized avoiding muscle relaxants. Current case differs because low-dose rocuronium plus sugammadex was used when relaxation was required.
Sungur Ulke et al. [12]	Prospective/clinical series	10 patients with MG, mean age 31 (+/- 12 yrs)	Videothoracoscopic-assisted thymectomy	Not fully detailed in accessible abstract. All patients were on pyridostigmine	Evaluated rocuronium–sugammadex strategy in MG patients undergoing thymectomy; neuromuscular monitoring used	Rocuronium used and reversed with sugammadex	Reported as an evaluation of feasibility of rocuronium/sugammadex in MG thymectomy	Strongly supports the approach used in the current case: reduced/controlled rocuronium use, monitoring, and sugammadex reversal.
Karlekar et al. [17]	Case report	17-year-old female, 90 kg, diagnosed with MG	Robot-assisted thoracoscopic thymectomy	Received prednisolone, pyridostigmine, and azathioprine on the day of surgery	Focused on robotic thymectomy challenges, including positioning, airway/ventilation, and hemodynamic monitoring	Not fully detailed in accessible abstract; NMBA not mentioned, reversal with neostigmine and glycopyrrolate	Stable ventilatory and hemodynamic parameters achieved after different maneuvers/techniques	Relevant because it highlights anesthetic concerns specific to minimally invasive/robotic thymectomy in MG, especially airway, positioning, and ventilation.
Pal et al. [18]	Case series	20 patients with MG, 8 male and 12 female, average age of 35 years	Robotic thymectomy	Pyridostigmine and corticosteroids were continued perioperatively	Reported perioperative and neuromuscular blockade considerations; notably described management without one-lung ventilation and without thoracic epidural	Small incremental doses of atracurium with neuromuscular monitoring, reversal with neostigmine and glycopyrrolate	Series presented as successful perioperative management of robotic thymectomy	Useful comparator for minimally invasive thymectomy in MG and supports individualized neuromuscular blockade planning.
Uchida et al. [13]	Case report	57-year-old female with subclinical MG, anti-AChR antibody positive, no neurological symptoms	Extended thymectomy	Subclinical MG identified preoperatively	Total intravenous anesthesia (TIVA) using propofol and remifentanil; depth of anesthesia was monitored using the Bispectral Index (BIS); postoperative epidural analgesia	Minimum rocuronium administered with TOF monitoring when needed; prolonged rocuronium effect observed, but TOF recovered to 100% before extubation. No reversal was given	Successful extubation; authors emphasized uncertainty of rocuronium sensitivity even in subclinical MG	Highly relevant because it supports the cautious use of minimal rocuronium under TOF monitoring, similar to the current case.
No et al. [14]	Retrospective comparative study	180 adult MG patients undergoing VATS thymectomy; 83 received sugammadex and 88 received neostigmine/glycopyrrolate	VATS thymectomy	Institutional retrospective cohort	Induction with propofol and remifentanil; neuromuscular blockade with rocuronium	Sugammadex group compared with neostigmine/glycopyrrolate group	patients who received sugammadex had significantly shorter postoperative hospital stays than those who received neostigmine	Provides larger-scale support for sugammadex use after VATS thymectomy in MG and strengthens the rationale for sugammadex in the current case.
Hien et al. [10]	Prospective observational study	90 patients with MG undergoing thoracoscopic thymectomy; 45 received propofol TCI, 45 received sevoflurane	Thoracoscopic thymectomy	Anticholinesterase and steroid therapy were stopped on the morning of surgery and restarted the next day	Compared propofol target-controlled infusion or sevoflurane anesthesia without muscle relaxant	No muscle relaxant technique	Prospective evidence supporting thoracoscopic thymectomy without muscle relaxant; Sevoflurane achieved higher levels of intraoperative muscular relaxation than propofol	Supports the principle that thymectomy in MG can often be performed without NMBA; current case adds that low-dose rocuronium with sugammadex is an option when relaxation is required.

Overall, this case emphasizes that anesthetic management of MG should be individualized according to disease severity, bulbar and respiratory symptoms, surgical requirements, and available monitoring. Avoidance of neuromuscular blocking agents remains preferable whenever feasible. However, when muscle relaxation is required, reduced-dose rocuronium with quantitative neuromuscular monitoring and sugammadex reversal represents a practical and effective strategy. Preoperative optimization, multidisciplinary planning, careful extubation criteria, and postoperative monitoring are essential to reduce the risk of respiratory complications and myasthenic crisis.

## Conclusions

The anesthetic management of patients with myasthenia gravis undergoing thoracoscopic thymectomy requires careful preoperative optimization, individualized anesthetic planning, and close postoperative monitoring. Avoidance of neuromuscular blocking agents remains preferable whenever feasible because of the increased sensitivity of these patients to non-depolarizing agents and the risk of prolonged weakness. However, when muscle relaxation is required to facilitate thoracoscopic surgical conditions, cautious use of low-dose rocuronium under quantitative neuromuscular monitoring, followed by sugammadex reversal, may be a reasonable approach. This case highlights the importance of multidisciplinary planning, preoperative intravenous immunoglobulin therapy in selected patients, train-of-four monitoring, careful extubation criteria, and postoperative intensive care observation to reduce the risk of respiratory complications and myasthenic crisis.
